# Modulation of epithelial homeostasis by HPV using Notch and Wnt

**DOI:** 10.1016/j.tvr.2024.200297

**Published:** 2024-11-13

**Authors:** June See Chong, John Doorbar

**Affiliations:** Department of Pathology, University of Cambridge, Cambridge, CB2 1QP, UK

## Abstract

Highly conserved signalling pathways such as Notch and Wnt are essential in the regulation of differentiation and proliferation processes during adult tissue homeostasis. Human papillomaviruses (HPVs) have evolved with humans to manipulate these signalling pathways to establish a basal reservoir of infected cells by limiting HPV-infected keratinocyte differentiation whilst ensuring that differentiating cells are in a replication-competent state. Here, we focus on the canonical Notch and Wnt signalling pathways and their crosstalk to ensure cell-fate lineage determination during epithelial homeostasis. We then examine how HPVs use their E6 and E7 proteins to inhibit differentiation and maintain stem-like characteristics using Notch and Wnt in HPV-infected cells. Determining the functions of E6 and E7 in the maintenance of the infected cell reservoir, and the molecular crosstalk between Notch and Wnt is vital for our understanding of HPV persistence, and may represent an important factor in the development of therapeutic agents for HPV-associated disease.

Signalling pathways are vital in the generation of cellular and morphological diversity during animal development and adult tissue homeostasis [1]. Notch and Wnt are two complex pathways that monitor and regulate one another to control cell fate decisions [2]. They often have opposing effects on cell fate determination which control progression along a specific cellular lineage [3]. Human papillomaviruses (HPVs) have co-evolved with a range of different animal species including humans for millions of years [4]. HPVs are highly tissue-restricted as during evolution, they became adapted to specific epithelial niches and their local microenvironments and have hence developed distinct tissue tropisms [[5], [6], [7], [8]]. Lesion formation begins when HPV infects a basal stem cell in the epithelium through microabrasions on the skin surface (Fig. 1) [[9], [10], [11]]. The basal stem cell then proliferates and asymmetrically divides to generate an array of transit amplifying cells and establish a reservoir of virally infected cells, and at a specific cell density allows some infected cells to commit to differentiation (Fig. 1). To ensure a replication-competent environment for genome amplification and packaging of viral particles, HPV uses E6 and E7 proteins for both episomal genome maintenance and the reactivation of cell division among the infected, differentiated cells [[12], [13], [14], [15]].

**Fig. 1:**
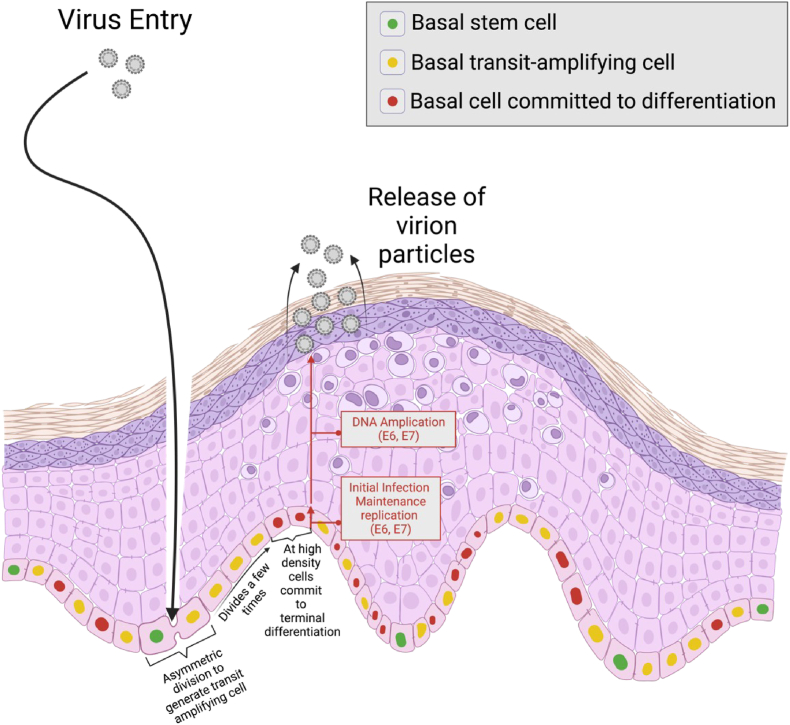
Events during HPV infection in the stratified squamous epithelium. HPV enters the multi-layer squamous epithelium through microabrasions on the skin surface and is thought to infect a basal stem cell. The infected basal stem cell asymmetrically divides to generate transit amplifying cells that undergo a fixed number of divisions prior to differentiation. At a specific cell density, they commit to differentiation and extend into the parabasal layers and eventually HPV virion particles are released from the stratified epithelium. The E6/E7 proteins modulate the timing of differentiation of cells in the epithelial basal layer.

HPV types from different genera use their E6 and E7 proteins to manipulate highly conserved signalling pathways to cause persistent papillomas [[16], [17], [18], [19], [20]]. In high-risk Alpha HPV types, E6 and E7 proteins drive cell proliferation in the basal and parabasal layers. High-risk E6 degrades p53 and PDZ substrates to inhibit differentiation and induce keratinocyte immortalization; high-risk E7 degrades pRb family members including p105 and p107 which control cell cycle entry in the basal layer, and p130 which controls cell cycle entry in the upper epithelial layers [[20], [21], [22], [23]]. Deregulated expressions of E6 and E7 in these types, in particular HPV 16 and 18, cause nearly all cases of cervical cancer and anal cancer as well as being a cause of oropharyngeal cancer [[24], [25], [26], [27], [28]]. During the normal productive cycle of these and other HPV types, E6 and E7's key function is play a role in homeostasis and enforce genome amplification. While low-risk HPVs cannot promote E6-dependent degradation of p53 or PDZ proteins, and low-risk E7 has lower affinities for p105 and 107 than its high-risk counterpart, persistent infection can still progress into problematic pathologies, such as papillomatosis and sometimes even cancers [[29], [30], [31], [32]]. For example, HPV type 11 causes recurrent respiratory papillomatosis in children which has no effective treatment. Its recurrent nature, despite surgical removal, blocks the lower airways and lungs, resulting in significant morbidity and occasional mortality [33,34]. Hence, the primary function of E6 and E7 of all papillomaviruses is to establish a viral reservoir by restricting basal keratinocyte differentiation whilst driving differentiating cells into a replication-competent state [35,36].

Here, we review the modulation of Notch and Wnt signalling pathways by the representatives of the Alpha high-risk and low-risk groups-types 16 and 11 respectively for persistent viral infection which can cause HPV-associated diseases. First, we review our understanding of the Notch and Wnt pathways in animal development, specifically the cell fate decisions required to maintain epithelial homeostasis. Then, we reflect on how HPVs have modulated the Notch and Wnt pathways to ensure viral persistence of the infected cell in the basal layer.

## 1 Role of Notch in epithelial and HPV-modulated homeostasis

**Fig. 2:**
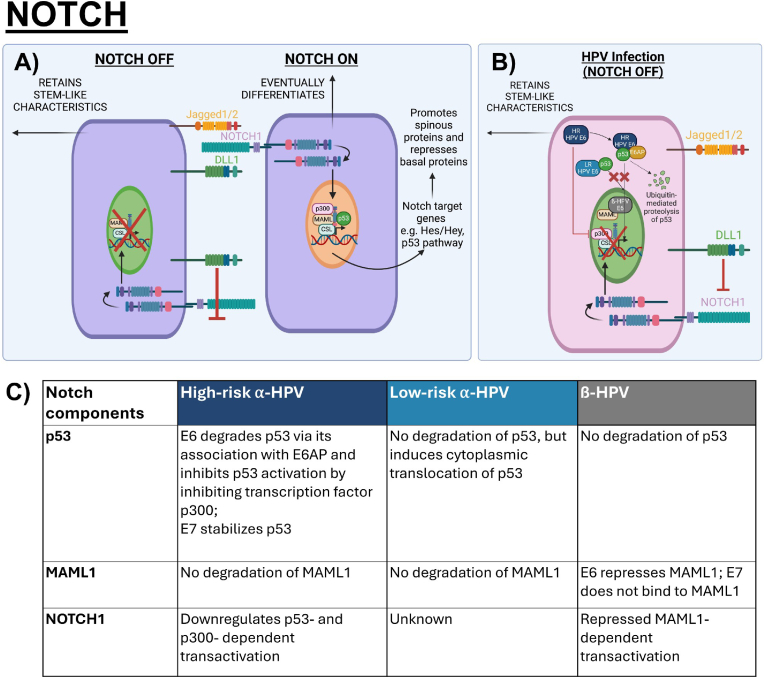
Canonical Notch signalling in epithelial homeostasis and HPV-modulated homeostasis. A) Notch activation for the commitment switch to differentiation from the basal-parabasal junction can be activated by Notch ligands Jagged1, Jagged2 or DLL1 to stimulate expression of the Hes/Hey families and its targets. The normal role of p53 also activates Notch signalling by binding to the Notch1 promoter. High-DLL1-expressing basal keratinocyte cis-inhibits Notch signalling to retain stem-like characteristics while inducing Notch1 activation and differentiation in neighbouring cell [43,52,57]. B) To delay differentiation in HPV-infected cells, different genera of HPVs use E6 to inhibit the Notch signalling pathway. High-risk Alpha HPV types degrade p53 using the E6/E6AP complex and further inhibit p53 activity by inhibiting transcription factor p300 to inhibit Notch1 transcription, low-risk types cannot degrade p53 but prevent its nuclear translocation and interaction with Notch target genes, whilst Beta HPVs bind to MAML to repress NICD activation [57,61,63,64]. C) Table summarizes the interactions of Notch components, including p53, MAML1 and NOTCH1, with Alpha- and Beta- HPV E6 and E7.

Canonical Notch signalling occurs when a cell-cell interaction between a Notch receptor and its ligands, Delta and Serrate, releases the intracellular domain of Notch (NICD) for nuclear translocation [[37], [38], [39]]. In the nucleus, NICD interacts with transcription factor CBF1/Su(H)/LAG-1 (CSL) to recruit coactivators, such as MAML proteins, to drive the transcription of Notch target gene families- HES and HEY which are associated with differentiation processes in the cervix and uterine endometrium, and the interfollicular epidermis [[40], [41], [42], [43]]. Canonical Notch signalling has been shown to function as the commitment switch from a proliferative basal cell to a terminally differentiated parabasal cell where it represses basal markers such as CK5 and CK14 and promotes spinous differentiation markers such as CK1, CK10 and IVL [[44], [45], [46]]. The Notch ligands detected in the epidermis that can activate Notch signalling are Delta-like 1 (DLL1), Jagged1 and Jagged2 which have variable expression patterns across the epithelial layers and distinct ligand-specific functional effects on cell fate determination. Jagged1 is primarily expressed in the parabasal layers and Jagged2 is expressed in the basal layer [47,48]. Both have been found to induce basal keratinocyte differentiation, but some studies have also functionally linked Jagged1 to increasing stem-like characteristics in cancer cells [47,[49], [50], [51]]. DLL1 expression is highest in basal stem cell clusters of healthy fetal and adult human epidermis, where high-DLL1-expressing basal keratinocytes were unresponsive to Notch activation and retained stem-like characteristics yet induced Notch1 activation and differentiation in neighbouring cells (Fig. 2A) [[52], [53], [54], [55]]. Tumour suppressor p53 can also target Notch signalling by binding to and transactivating the Notch1 promoter, resulting in keratinocyte commitment to differentiation [56,57].

Across different genera of HPVs, the Notch pathway is a common target of the E6 protein, emphasizing its important deregulation to ensure viral persistence and a HPV-modulated homeostasis [58,59]. Moreover, during the progression of cervical cancer, down-modulation of Notch1 is required for sustained transcription of the E6/E7 genes and subsequent malignant transformation [60]. For the high-risk Alpha HPV types, E6-dependent degradation of p53 with E6AP downregulates p53-dependent transactivation of the Notch1 promoter, resulting in deregulated differentiation. Moreover, high-risk E6 inhibits transcriptional activity of co-activator p300, which inhibits p53 and Notch1 activation [61,62]. For Beta HPV types, repressed transactivation of the Notch signalling occurs through inhibition of MAML1-a coactivator of Notch signalling, resulting in delayed differentiation (Fig. 2B and C) [57,58,63]. Although low-risk Alpha types cannot degrade p53, their E6 proteins have been found to induce cytoplasmic translocation of p53, hence interfering with its nuclear function [29,64]. The role of the E7 protein on the Notch pathway in disrupting differentiation is less clear, but it is reported that HPV16 E7 stabilizes p53, suggesting the possibility that high-risk E7 may rescue Notch1 expression [[65], [66], [67], [68]]. β-genus E7 does not bind to MAML1 and regulate Notch1 expression, but other critical associations between E7 and Notch targets that drive keratinocyte differentiation have yet to be fully explored.

## 2 Role of Wnt in epithelial and HPV-modulated homeostasis

**Fig. 3:**
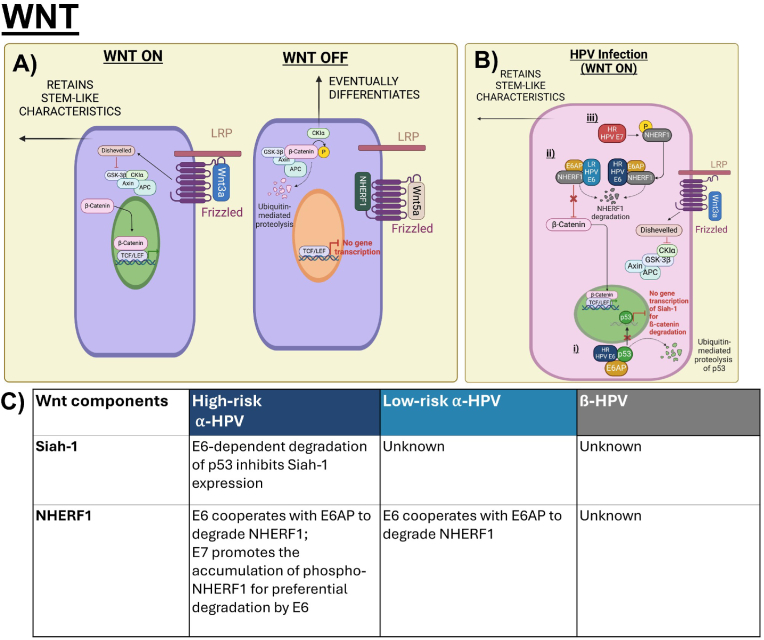
Canonical Wnt/β-catenin signalling in epithelial homeostasis and HPV-modulated homeostasis. A) Signalling cells secrete canonical Wnt ligands, such as Wnt3a, to activate the Wnt/β-catenin signalling which promotes self-renewal and inhibits differentiation in stem cells. Non-canonical Wnt5a ligand and tumour suppressor NHERF1 bind to Frizzled, thereby inhibiting Wnt3a-dependent LRP phosphorylation and inhibiting accumulation of β-catenin. Inhibition of canonical Wnt signalling has been reported to induce epithelial cell differentiation [79,82,85]. B) HPVs use E6 and E7 to increase canonical Wnt/β-catenin signalling. i) High-risk E6-dependent degradation of p53 prevents the transcriptional activation of ubiquitin ligase Siah-1 which degrades β-catenin; ii) High and low-risk E6 cooperate with E6AP to degrade NHERF1 which subsequently activates the Wnt/β-catenin signalling pathway; iii) High-risk E7 increases the accumulation of phosphorylated NHERF1 which is preferentially targeted by E6 for degradation [89,90,94]. C) Table summarizes the interactions of Wnt components, including Siah-1 and NHERF1, with Alpha- and Beta- HPV E6 and E7.

The canonical Wnt/β-Catenin signalling pathway is activated by the secreted Wnt1 class ligands, including Wnt2, Wnt3, Wnt3a and Wnt8a, which are released by signalling cells, that bind to Frizzled receptors (FZD1-10) and LRP5/6 proteins which recruits Dishevelled to inhibit the Axin destruction complex, hence allowing the accumulation of cytoplasmic β-catenin and subsequent nuclear translocation [69,70]. In the nucleus, β-catenin binds with transcription factors and recruits co-activators to drive the expression of target genes, such as *Axin2* and *c-Myc*, to facilitate cell proliferation, survival, differentiation and migration [[71], [72], [73]]. The pathway has been reported to regulate stem cell clusters by safeguarding epigenetic stability and controlling cell proliferation whereas during oncogenesis, β-Catenin expression is deregulated in cancer stem cells [[74], [75], [76]]. The canonical Wnt pathway is highly conserved and can only be activated via the binding of the secreted canonical Wnt1 class ligands, such as Wnt3a (Fig. 3A) [77,78]. Like Notch, Wnt ligands also exhibit different functional effects on proliferation and differentiation. Wnt3a has been found to rescue self-renewal and inhibit differentiation in epidermal, embryonic and haematopoietic stem cells [[79], [80], [81]]. As negative regulators of canonical Wnt signalling, noncanonical ligand Wnt5a and tumour suppressor NHERF1 compete with Wnt3a for the binding to Frizzled. This promotes the degradation of β-catenin and inhibits ß-catenin-dependent Wnt signalling, subsequently inducing differentiation in stem cells (Fig. 3A) [[82], [83], [84], [85]].

To reduce commitment of differentiation and maintain self-renewal processes of epidermal stem cells, high- and low-risk HPV E6 proteins have been found to upregulate the β-catenin/TCF signalling response which regulate genes involved in cell polarity, proliferation, migration, and differentiation like *c-myc*, *Cyclin D1*, and *Axin-2* [72,[86], [87], [88]]. High-risk E6 cooperates with E6AP to induce p53 degradation, which inhibits the expression of ubiquitin ligase Siah-1 that degrades β-catenin (Fig. 3B). Loss of p53 and subsequent loss of Siah-1 expression prevents the degradation of β-catenin and allows for its accumulation (Fig. 3B) [89]. Low-risk E6 proteins do not degrade p53, but both high and low-risk E6 cooperate with E6AP to degrade NHERF1- a highly conserved PDZ protein to activate Wnt signalling [86,90,91]. NHERF1 is tumour-suppressive and regulates cellular processes of differentiation and inhibits cervical cancer cell proliferation, thus HPV degrades NHERF1 to delay differentiation and augment proliferation [92,93]. Although the specific roles of E7 in Wnt signalling have been less well-characterized, it has been suggested that high-risk E7 promotes the accumulation of phosphorylated NHERF1 for preferential degradation by E6 (Fig. 3B and C) [94]. Some studies also suggest E7 may contribute to β-catenin stabilization in the cytoplasm where E7 may bind to PP2A-a phosphatase that induces β-catenin degradation [95,96].

## 3 Role of Notch & Wnt in epithelial and HPV-modulated homeostasis

Crosstalk between the Notch and Wnt signalling pathways where they directly and indirectly regulate each other is vital in many developmental processes. This is particularly important in the process of cell fate determination and cell lineage [2,3,97]. For example, Notch promotes the differentiation of epithelial cells by inhibiting the Wnt signal that promotes their self-renewal [98].

**Fig. 4:**
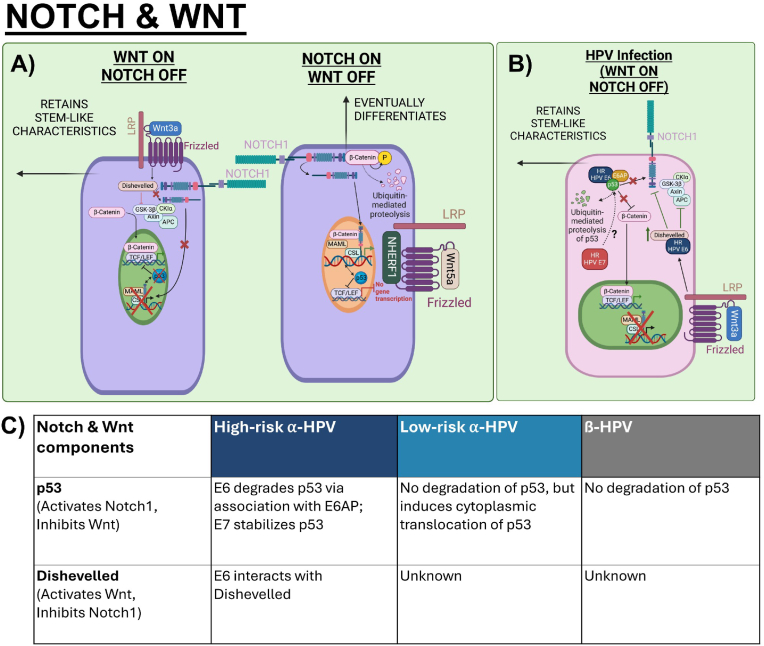
Crosstalk of Notch and Wnt in epithelial homeostasis and HPV-modulated homeostasis. A) Notch and Wnt regulate one another to achieve Wnt-ON/Notch-OFF or Notch-ON/Wnt-OFF states. For Wnt-ON/Notch-OFF, Dishevelled interacts with Notch1 and inhibition of NICD-dependent transcription of Notch target genes leads to loss of p53. Loss of p53 subsequently enables accumulation and activation of β-catenin. For Notch-ON/Wnt-OFF, the Notch1 receptor sequesters β-catenin at the membrane. Moreover, NICD can form a complex with β-catenin and CSL in the nucleus to deactivate β-catenin. p53-dependent activation of Notch and inhibition of β-catenin induces the cell to commit to differentiation [99,104,105]. B) HPV's modulation on the crosstalk between Notch and Wnt depends on mutual effectors in the pathways. High-risk E6 degrades p53 which has an activating function on Notch1 and inhibitory function on β-catenin. High-risk E6 also binds to and stabilizes Dishevelled which inhibits Notch1 and activates Wnt by inhibiting the Axin destruction complex. The role of E7 and its cooperative effects with E6 on the crosstalk remain to be well-characterized [108,109]. C) Table summarizes the interactions of Notch & Wnt components, including p53 and Dishevelled, with Alpha- and Beta- HPV E6 and E7.

Since both signalling pathways regulate proliferation and differentiation, strict controls of either Notch-ON/Wnt-OFF or Notch-OFF/Wnt-ON are required to avoid conflicts between the two signalling pathways. Notch downregulates Wnt signalling by promoting the degradation of β-catenin to promote differentiation of epidermal stem cells and inhibit their self-renewal processes whilst Wnt inhibits Notch to control cell-fate specification during epidermal development [[98], [99], [100]]. It is suggested that most convergence of the pathways rely on common transcriptional targets, but direct interactions between the pathways have also been reported (Fig. 4A) [[101], [102], [103]]. To achieve Notch-ON/Wnt-OFF, Notch1 can antagonize activated β-catenin activity by isolating β-catenin at the membrane, and active NICD can form a complex with β-catenin via CSL in the nucleus, hence deactivating β-catenin [104]. To achieve Wnt-ON/Notch-OFF, Dishevelled directly interacts with the carboxyl terminal of Notch1 and inhibits CSL transcription factors to antagonize Notch signalling [99,100]. Besides direct interactions between Notch and Wnt, an overlapping player between the pathways could be tumour suppressor p53. Notch1 and p53 are co-regulators of each other in epithelial cells whereas p53 downregulates β-catenin activation by inducing expression of ubiquitin ligase Siah-1 that degrades β-catenin [56,57,105,106]. However, the exact mechanisms between these molecular interactions using p53 remain to be clearly elucidated.

Understanding how HPV modulates the Notch and Wnt pathways independently is only the first step in learning how HPV orchestrates the signalling pathways to maintain a HPV-modulated homeostasis. First, we consider how HPVs use E6 to alter key effectors involved in both pathways (Fig. 4B and C). For example, p53 has a dual function in activating Notch1 and inhibiting Wnt [23,57,107]. Hence, high-risk E6 degradation of p53 results in inhibition of Notch and upregulation of Wnt [86,108]. Moreover, high-risk E6 also targets NHERF1 for degradation, further enhancing Wnt [90]. Another example is Dishevelled which enables β-catenin accumulation and inhibits the Notch1 receptor. HPV16 E6 has been found to interact with Dishevelled, therefore augmenting Wnt and perhaps inhibiting Notch [109,110]. Second, we examine how E7 alters key effectors during the crosstalk between Notch and Wnt. The functions of E7 in Notch and Wnt are less established, but high-risk E7 may stabilize p53 hence increasing p53 transactivation of the Notch1 promoter and Siah-1 expression that degrades B-catenin.

The roles of highly conserved Notch and Wnt signalling pathways in epithelial homeostasis are well defined; they are essential for cell fate determination and regulating differentiation and proliferation processes. HPVs use their E6 and E7 proteins to disrupt Notch and Wnt signalling independently as well as interfering with their crosstalk to ensure the maintenance of a viral reservoir in the basal layer. However, it is important to consider that significant variations of E6 and E7 expression levels exist between cells due to a fine balance between splicing factors and cis-regulatory RNA elements on HPV pre-mRNA [111,112]. The varying levels of E6 and E7 expression in individual cells may affect its level of regulation on Notch and Wnt signalling, which may give rise to the heterogeneity required for effective signalling. Further understanding of how the levels of E6 and E7 affect the Notch and Wnt pathways which impact differentiation and proliferation processes of HPV-infected cells is critical. Determining the functions of E6 and E7 alone and in cooperation with each other during this molecular crosstalk between Notch and Wnt is vital for our understanding of HPV persistence. Key players in this crosstalk that are modulated by E6 and E7 have yet to be well-characterized; p53 and Dishevelled hold promise whilst new players may yet to be revealed, representing an important consideration in the development of targeting agents for HPV-associated disease.

## CRediT authorship contribution statement

**June See Chong:** Writing – original draft, Visualization, Investigation, Formal analysis, Data curation, Conceptualization. **John Doorbar:** Writing – review & editing, Supervision, Resources.

## Declaration of competing interest

The authors declare that they have no known competing financial interests or personal relationships that could have appeared to influence the work reported in this paper.

## Data Availability

No data was used for the research described in the article.
